# Cortical capillary recruitment by rosuvastatin in experimental brain trauma is associated with increased NO production

**DOI:** 10.1186/cc10921

**Published:** 2012-03-20

**Authors:** P Bentzer, M Jungner

**Affiliations:** 1 Lund University, Lund, Sweden

## Introduction

Microvascular dysfunction, characterized by edema formation secondary to increased blood-brain barrier (BBB) permeability and decreased blood flow, contributes to poor outcome following brain trauma. Recent studies have indicated that statins may counteract edema formation following brain trauma but little is known about other circulatory effects of statins in this setting. The objective of the present study was to investigate whether statin treatment improves brain microcirculation early after traumatic brain injury, and whether microvascular effects are associated with altered production of nitric oxide and prostacyclin.

## Methods

After fluid percussion injury, rats were randomized to intravenous treatment with 10 mg/kg rosuvastatin or vehicle. Brain edema (wet/dry weight), BBB integrity (^51^Cr-EDTA blood to brain transfer), cerebral blood flow (^14^C-iodoantipyrine autoradiography), and the number of perfused cortical capillaries (FITC-albumin fluorescence microscopy) were measured at 4 and 24 hours. Production of NO and prostacyclin was estimated by measuring the stable degradation products nitrite and nitrate (NO_x_), and 6-keto-PGF-1α in plasma. Sham injured animals were treated with vehicle and analyzed at 4 hours.

## Results

Trauma resulted in brain edema, BBB dysfunction, and reduced cortical blood flow, and no effect of treatment on these parameters could be detected. Trauma also induced a reduction in the number of perfused capillaries, which was improved by statin treatment. Statin treatment led to increased plasma NO_x _levels and reduced mean arterial blood pressure. The 6-keto-PGF-1α levels tended to increase after trauma, and were significantly reduced by rosuvastatin.

## Conclusion

Rosuvastatin treatment improves microcirculation after traumatic brain injury by increasing the number of perfused capillaries. This effect is associated with increased NO and reduced prostacyclin production.
